# L-Theanine Ameliorates Obesity-Related Complications Induced by High-Fat Diet in Mice: Insights from Transcriptomics and Metabolomics

**DOI:** 10.3390/foods13182977

**Published:** 2024-09-19

**Authors:** Zhaofeng Du, Guohuo Wu, Huijun Cheng, Tingting Han, Daxiang Li, Zhongwen Xie

**Affiliations:** 1 State Key Laboratory of Tea Plant Biology and Utilization, School of Tea and Food Sciences and Technology, Anhui Agricultural University, Hefei 230036, China; zfd2001@fynu.edu.cn (Z.D.); wuguohuo@fynu.edu.cn (G.W.); c_huijun@126.com (H.C.); tings_han@163.com (T.H.); dxli@ahau.edu.cn (D.L.); 2 Joint Research Center for Food Nutrition and Health of IHM, Anhui Agricultural University, Hefei 230036, China; 3 Engineering Technology Research Center of Anti-Aging Chinese Herbal Medicine of Anhui Province, School of Biology and Food Engineering, Fuyang Normal University, Fuyang 236041, China; 4 College of Biological Sciences and Technology, Yili Normal University, Yining 835000, China

**Keywords:** L-theanine, obesity-related complications, metabolites homeostasis, transcriptomics, functional food

## Abstract

Obesity is a major public health concern globally. Plant-based ingredients have been proposed as alternative treatments for obesity. L-Theanine (THE), a unique nutraceutical component of tea, is known for its neuroprotective and cognitive benefits. However, there are few reports on THE’s effects and mechanisms in improving obesity and its complications. In this study, the alleviating effects and potential mechanisms of THE on obesity-related complications (ORCs) induced by a high-fat diet(HFD) in mice were explored by performing biochemical, hepatic transcriptomics, and plasma metabolomics analyses. The results indicated THE (900 mg/kg of body weight) was effective in mitigating ORCs by decreasing body weight gain and fat deposition, improving glycolipid metabolism disorders, inflammation dysregulation, and alleviating fatty liver formation due to long-term HFD. The hepatic transcriptomics data suggested that THE intervention suppresses the lipid metabolism and inflammation pathways in HFD-fed mice, thereby inhibiting hepatic steatosis and inflammation. Moreover, plasma metabolomics analysis revealed that THE exhibited positive effects on the homeostasis of plasma metabolite balance, such as phosphatidylcholine (PC(14:0/18:1)), phosphatidylethanolamine (Lyso-PE(14:0)), phosphatidic acid (PA(16:0e/18:0)), stigmasterol, and deoxycholic acid glycine conjugate. These metabolites were strongly correlated with ORC-related indicators. Our results indicated that THE, as a functional food additive, possesses potential for ORC alleviation. However, the exact molecular mechanism of how THE alleviates ORCs needs to be investigated in the future.

## 1. Introduction

Due to the changes in human dietary patterns and lifestyle, excessive intake of high calorie foods and inactivity result in fat accumulation and obesity. Obesity is a chronic epidemic of global concern and has become one of the serious problems facing global public health in the 21st century. The World Health Organization (WHO) defines adults with a body mass index (BMI) exceeding 25 kg/m^2^ as overweight, and those with a BMI exceeding 30 kg/m^2^ as obese. In recent decades, the prevalence of overweight and obesity has sharply increased. According to the World Obesity Atlas 2023, the overweight and obesity rate among the global population aged over 5 will rapidly increase from 38% in 2020 to 51% in 2035, with the number of people rising from 2.6 billion to over 4 billion, including nearly 2 billion obese people [1]. Researchers have shown that obesity is an important risk factor for a series of complications, including fatty liver, diabetes, hypertension, and cardiovascular disease [2,3], which seriously threaten human health, and these complications are collectively referred to as obesity-related complications (ORCs).

Nowadays, the development of chemical drugs for treating ORCs has made significant progress. Lipid-lowering drugs like statins and ezetimibe significantly lower the incidence rate of coronary heart related diseases and are the first choice among those who want to reduce their cholesterol levels [4,5,6]. However, epidemiological studies have shown that taking statins or ezetimibe also has many side effects, including muscle toxicity, mitochondrial dysfunction, and hepatotoxicity. Among these, myocardial toxicity is the main reason for the limited use of statins [7,8,9,10]. Aside from the difficulty of adhering to long-term exercise, as well as the side effects associated with anti-obesity pharmaceuticals [11], finding effective, safe, and easily adhered to alternative strategies for ORC relief is imperative.

Dietary therapy is being proposed as a potential alternative for ORCs [12]. Researchers have shown that natural products derived from plants have effective preventive and potential therapeutic effects on glycolipid metabolism disorders and have low side effects and usage costs [13,14]. People have been using natural plant-based ingredients to prevent and treat metabolic diseases for a long time, and phytomedicine has made significant contributions to the history of human disease resistance. Modern research has shown that phytomedicine can prevent and treat diseases, which are directly related to the active functional ingredients contained in plants. Natural plant polyphenols, flavonoids, and polysaccharides have been proven to have good hypoglycemic and lipid-lowering activities [15,16,17,18], and they have broad application prospects in obesity prevention and treatment. Therefore, the development of efficient natural plant extracts to prevent ORCs has significant potential and application value.

L-Theanine (THE) is a unique non-protein amino acid found in tea that is known for its various health benefits, including calming the nerves, anti-depression, improving cognition, sleep, and immune regulation properties [19,20]. Additionally, THE also has a positive effect on the body’s nutritional metabolism. Lin et al. demonstrated that THE can regulate glucose, lipid, and protein metabolism by activating insulin and AMPK-related pathways [21]. Peng et al. found that THE activates AMPK and induces browning and thermogenesis of white adipose tissue in the groin, thereby improving insulin sensitivity in mice [22]. Zheng et al. reported that THE reduces the levels of triglycerides (TG) and free fatty acids in mice serum [23]. Given these findings, it is reasonable to hypothesize that THE could be a functional food additive for alleviating ORCs. However, there are few reports on the improvement effect and mechanism of THE as a food additive on ORCs. Thus, this study aimed to investigate the alleviating effects and mechanisms of THE on ORCs induced by long-term HFD via biochemical, hepatic transcriptomics, and plasma metabolomics analyses. This research seeks to offer theoretical support for THE as a functional food additive.

## 2. Materials and Methods

### 2.1. Animals Experiment

C57BL/6J male mice (5 weeks old, weight 20.4 ± 0.23 g) free of specific pathogens, were purchased from Gem Pharmatech Co., Ltd. (Nanjing, China) and were housed in cages at the laboratory animal facility center of Anhui Agricultural University. All cages were maintained under controlled environmental conditions (temperature (22 ± 1 °C) and humidity (50 ± 5%) under a 12:12 h light/dark cycle), with food and water ad libitum. After 1 week of acclimatization, mice were divided into four groups (*n* = 6 per group) as follows: low fat-diet group (LF), high fat-diet group (HF), HF + low dose of THE group (THE, 300 mg/kg), and HF + high dose of THE group (THE, 900 mg/kg). All diets (details are described in Table S1) were purchased from Trophic Animal Feed High-Tech Co., Ltd. (Nantong, China). The THE-treated group mice received THE by gavage once daily for 26 weeks. And the LF and HF group mice were given the equal amount of water. THE (purity of 98%) was obtained from Nanjing Herb Source Bio-technology Co., Ltd. (Nanjing, China), and an aqueous solution of THE was prepared freshly every day. All animal experimental procedures were approved by the Institutional Animal Care and Use Committee of Anhui Agricultural University (approval no. AHAU 2016-028).

### 2.2. Sample Collection

In each group, after 26 weeks of treatment, all mice were fasted for 12 h, anesthetized with 4% chloral hydrate (10 mL/kg, i.p.), and sacrificed. Blood was collected from the ophthalmic vein. Plasma was centrifuged at 3000 r/min for 5 min at 4 °C and stored at −80 °C. On a scale, liver and abdominal fat weights were measured. A piece of liver tissue was immediately frozen in liquid nitrogen and stored at −80 °C for gene expression or transcriptomic analysis. The rest of the liver tissue was fixed in formaldehyde solution for the histological experiments.

### 2.3. Biochemical Indexes Analysis

Fasting blood glucose levels were determined using Glu-test strips (Nova Biomedical, Waltham, UK). Plasma insulin concentrations were measured using a commercial kit (Shanghai Jianglai, Shanghai, China). Plasma total cholesterol (T-CHO), triglyceride (TG), and low-density lipoprotein cholesterol (LDL-C) levels were measured using micro test kits (Nanjing Jiancheng, Nanjing, China). The enzymatic activities of alanine aminotransferase (ALT) and aspartate transaminase (AST) were analyzed using enzyme kits (Nanjing Jiancheng, Nanjing, China).

### 2.4. Hematoxylin–Eosin (HE) Staining and Analysis

HE staining was performed according to a previously published protocol [24]. Briefly, liver tissue samples were fixed in 10% neutral-buffered formalin, embedded in paraffin, and sectioned at 5 µm thickness. Sections were deparaffinized, rehydrated, and stained with hematoxylin and eosin subsequently. After dehydration and clearing, the sections were mounted with a coverslip. Images of the stained sections were captured using a LEICA DM500 microscope equipped with a LEICA ICC50 W camera (Wetzlar, Germany). The number of hepatic adipose infiltration cells was manually quantified using Image J2 software (National Institutes of Health, Bethesda, MD, USA). The analysis was performed on five randomly selected fields per section.

### 2.5. Quantitative Real-Time PCR

As described in a previous publication [25], real-time PCR was performed using SYBR Green Master Mix and the Real-Time PCR Detection System (CFX96 Touch, Bio-RAD, Hercules, CA, USA). A list of primer sequences used for this study is presented in Supplementary Table S2.

### 2.6. RNA Sequencing and Data Analysis

Sequencing of the liver transcripts was performed on three randomly selected liver samples from each of the LF group, HF group, and HF+THE-H (THE, 900 mg/kg) group. The TruSeq library was prepared as previous described [26]. Expression levels were indicated by fragments per kilobase of transcript per million fragments mapped (FPKM) for each sample. The raw sequence data were analyzed according to our previously published method [26]. Briefly, the DESeq 2 package (1.12.3) was used to analyze expression differences between groups. The Benjamini and Hochberg method was applied to adjust the resulting *p*-values to control the false discovery rate. Genes with DESeq-adjusted *p*-values of less than 0.05 were considered differentially expressed.

### 2.7. Plasma Metabolomics Analysis

Plasma metabolite extraction was performed as described in previous studies with a few modifications [27,28]. Briefly, 140 µL acetonitrile (including 5 μg/mL glibenclamide as an internal standard) was added to 20 μL of plasma and vortexed for 3 min. The mixture was then incubated in an ice bath for 20 min, followed by centrifugation at 16,000 r/min for 10 min at 4 °C. The supernatant was collected and filtered through a 0.22-micron organic membrane prior to metabolomic analysis. Metabolite analysis was conducted using high-resolution mass spectrometry (UHPLC-Orbitrap-MS/MS) (Thermo Fisher Scientific, Waltham, MA, USA). Four parallel experiments were carried out for each of the LF, HF, and HF+THE-H groups. Further details are provided in Supporting Text S1.

### 2.8. Statistical Analysis

Dataare presented as the mean ± SEM. Multiple group comparisons were conducted using one-way ANOVA or two-way ANOVA followed by Tukey’s test, as appropriate. Differences between two groups were assessed using the Student’s *t*-test. Statistical analyses were conducted using GraphPad Prism 5 software, and *p*-values less than 0.05 were considered statistically significant.

## 3. Results

### 3.1. THE Ameliorated Obesity Phenotype in HFD-Induced Obese Mice

**Figure 1:**
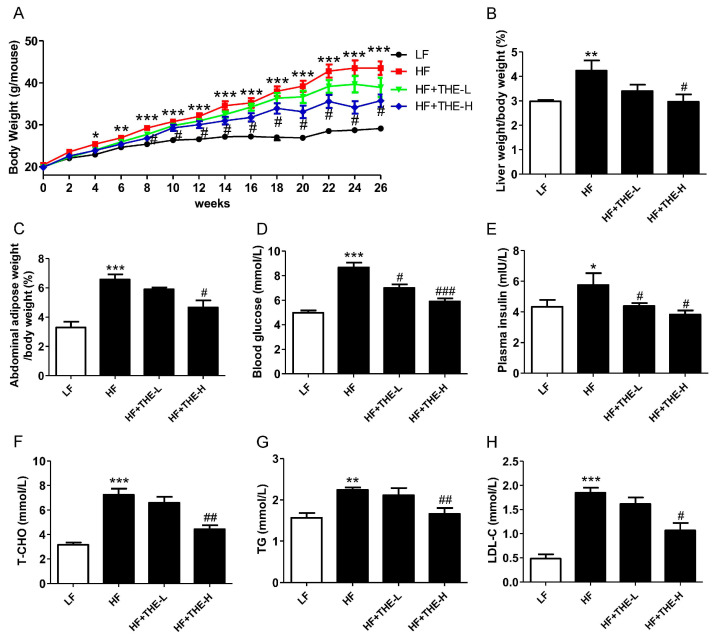
THE alleviated the obesity phenotype in HFD-induced obese mice. Notes: LF, low-fat diet group; HF, HFD group; HF+THE-L, HFD group mice with low-dose THE intervention; HF+THE-H, HFD group mice with high-dose THE intervention. (**A**) Body weight curves; (**B**) the ratio of liver to body weight; (**C**) the ratio of abdominal adipose weight to body weight; (**D**) fasting blood glucose; (**E**) plasma insulin; (**F**) total cholesterol; (**G**) triglyceride; (**H**) low-density lipoprotein cholesterol. * *p* < 0.05, ** *p* < 0.01, *** *p* < 0.001 versus LF group; # *p* < 0.05, ## *p* < 0.01, ### *p* < 0.001 versus HF group (*n* = 6, means ± SEM).

Obesity-related complications (ORCs) are characterized by obesity, insulin resistance, and dyslipidemia. According to Figure 1, mice in the HF group showed a marked increase in body weight, liver weight, and abdominal adipose weight (Figure 1A–C), as well as higher concentrations of plasma glucose, insulin, T-CHO, TG, and LDL-C (Figure 1D–H) compared to LF group mice. After a 26-week experiment, low-dose THE treatment significantly reduced elevated plasma glucose (by 7.85%) and insulin (by 23.67%) levels, but did not significantly affect body weight, liver weight, abdominal adipose weight, or plasma T-CHO, TG, and LDL-C levels compared to HF group mice. By contrast, high-dose THE treatment for 8 weeks significantly decreased the body weight of obese mice, with this preventive effect lasting until the end of the experiment. Additionally, after a 26-week experiment, the high-dose THE treatment significantly deceased liver weight, abdominal adipose weight, and attenuated the elevation of all observed plasma parameters, including glucose (by 31.92%), insulin (by 33.40%), T-CHO (by 38.80%), TG (by 26.13%), and LDL-C (by 42.26%), compared to continuous HF group mice (Figure 1B–H). 

### 3.2. THE Prevented Hepatic Steatosis in HFD-Induced Obese Mice

**Figure 2:**
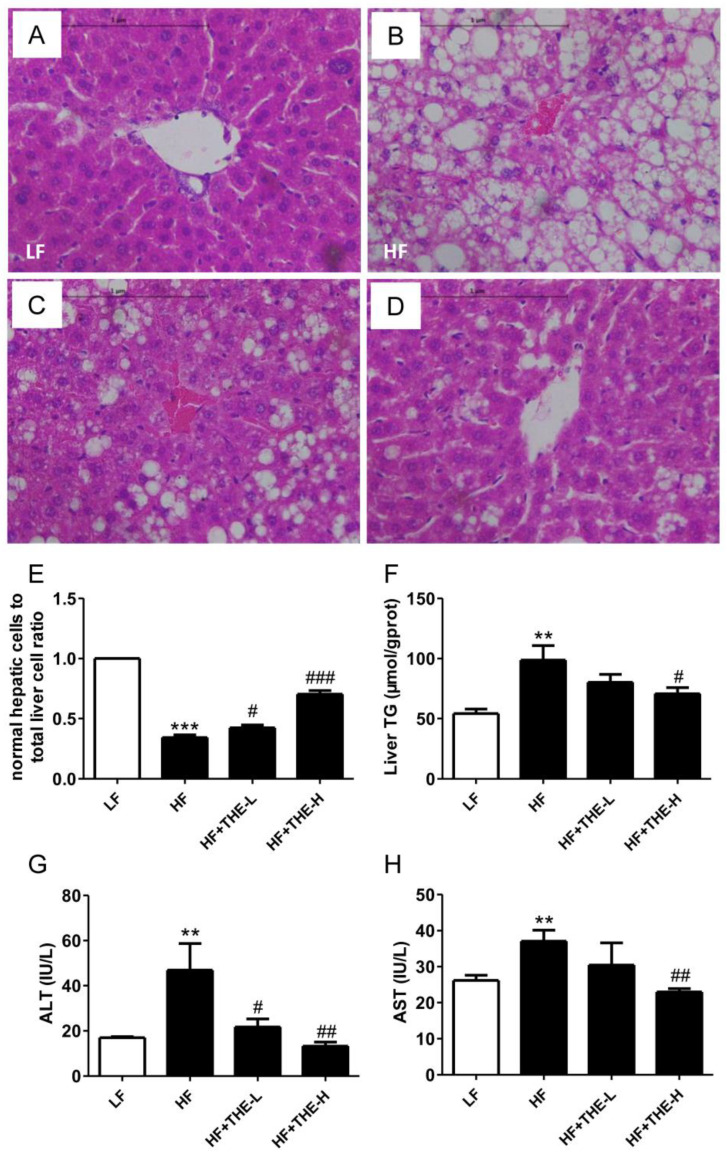
THE improved hepatic steatosis in HFD-induced obese mice. (**A**–**D**) (600 dpi resolution) Histological examination of liver structure with HE staining in different groups of mice; (**E**) normal hepatic cell-to-total liver cell ratio (statistics were conducted by randomly selecting 5 fields of view from each tissue section); (**F**) TG levels in liver; (**G**,**H**) the activities of serum ALT and AST, respectively. ** *p* < 0.01, *** *p* < 0.001 versus LF group; # *p* < 0.05, ## *p* < 0.01, ### *p* < 0.001 versus HF group (*n* = 6, means ± SEM).

Histopathological sections of liver tissue showed that mice in the HF group mice had abnormally fatty hepatocytes, whereas mice in the LF group displayed clear hepatic lobules without lipid droplets (Figure 2A,B). Treatment with THE restored normal liver architecture, with hepatocytes exhibiting reduced fat droplet deposition (Figure 2C,D). High-dose THE was more protective than low-dose THE. We also measured the normal liver cell-to-total hepatic cell ratio in the visual field. Figure 2E shows that THE-treated mice exhibited more normal liver cells than HF group mice. Additionally, high-dose THE significantly decreased the liver TG level and plasma ALT and AST activities in obese C57BL/6 mice (Figure 2F–H), whereas low-dose THE intervention significantly reduced only ALT activity. 

### 3.3. THE Supressed Liver Lipid Metabolism and Inflammation in HFD-Induced Obese Mice

**Figure 3:**
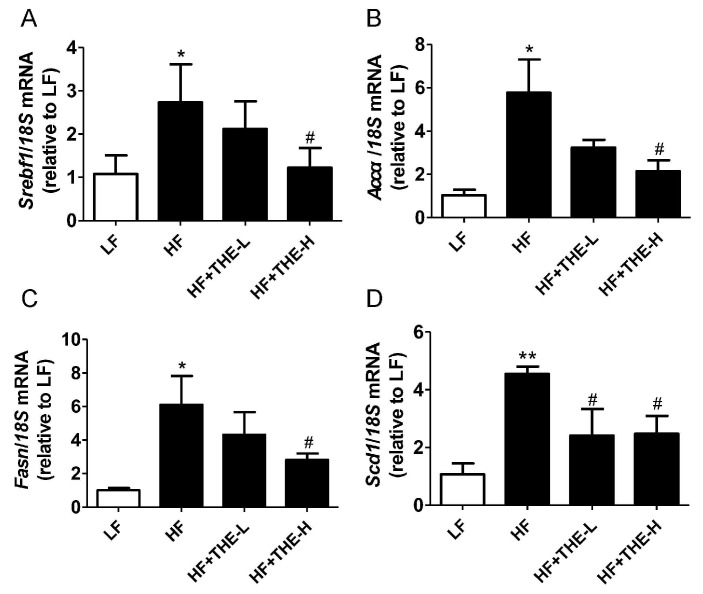
L-Theanine decreased the expression of hepatic lipogenesis-related genes in HFD-induced obese mice. (**A**) *Srebf1*; (**B**) *Accα*; (**C**) *Fasn*; (**D**) *Scd1*. * *p* < 0.05, ** *p* < 0.01 versus LF group; # *p* < 0.05 versus HF group (*n* = 6, means ± SEM).

To investigate the mechanisms by which THE alleviates ORCs, the expression of genes involved in lipogenesis and inflammation was analyzed in mouse liver tissues. Real-time PCR data showed remarkable increases in the expression of sterol regulatory element-binding transcription factor 1 (*Srebf1*), acetyl-CoA carboxylase α (*Accα*), fatty acid synthase (*Fasn*), and stearoyl-CoA desaturase (*Scd1*) in the livers of HF group mice (*p* < 0.05) (Figure 3A–D). Oral administration of high-dose THE significantly decreased the expression of these adipogenesis genes (all *p* < 0.05). However, low-dose THE significantly reduced only *Scd1* expression. 

**Figure 4:**
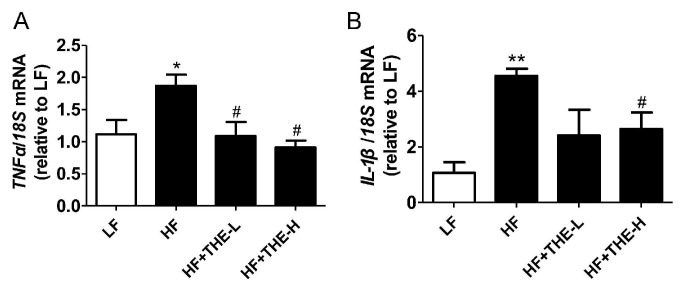

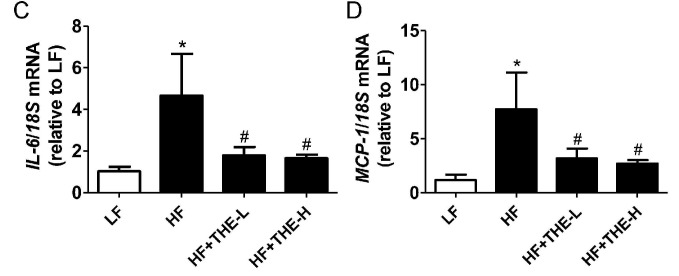
L-Theanine reduced the expression of inflammatory response genes in HFD-induced obese mice. (**A**) *TNFα*; (**B**) *IL-1β*; (**C**) *IL-6*; (**D**) *MCP-1*. * *p* < 0.05, ** *p* < 0.01 versus LF group; # *p* < 0.05 versus HF group (*n* = 6, means ± SEM).

Chronic inflammation is a major pathological factor for ORCs, and liver inflammation contributes to fatty liver disease [29]. We observed significant increases in the expression of pro-inflammatory cytokines *TNFα*, *IL-1β*, *IL-6*, and *MCP-1* in the livers of HF group mice compared to the LF group. High-dose THE resulted in a profound decrease in the expression of these cytokine genes in HFD mice liver tissue (Figure 4A–D). Low-dose THE significantly decreased the expression of *TNFα*, *IL-6*, and *MCP-1* genes but did not significantly reduce *IL-1β* expression compared to the HF group mice.

### 3.4. THE Altered Liver Lipid Metabolism and Inflammatory Pathways in Obese Mice Induced by HFD Revealed by RNA-seq Data

**Figure 5:**
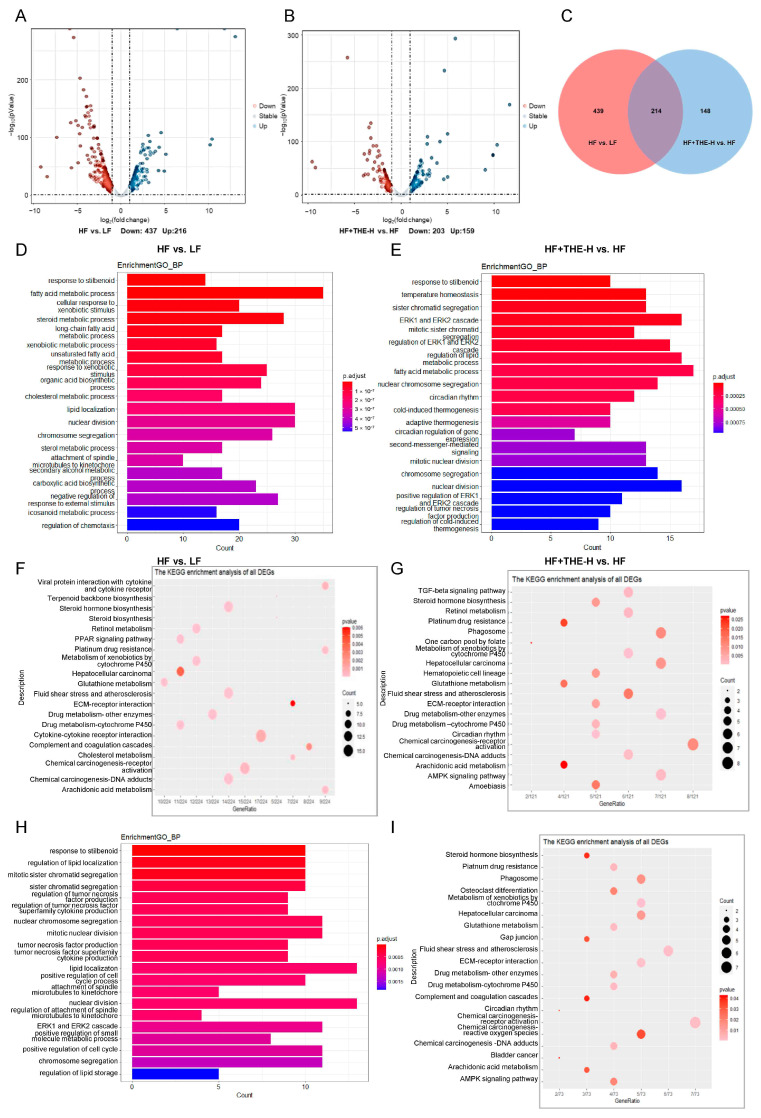
Functional enrichment analysis of differentially expressed genes in the liver tissues of various groups of mice ((**A**–**I**), 600 dpi resolution). (**A**) Volcano plot displaying DEGs between HF and LF; (**B**) Volcano plot displaying DEGs between HF+THE-H and HF; (**C**) Venn diagram of DEGs in different group of mice; (**D**) GO enrichment analysis of DEGs between HF and LF; (**E**) GO enrichment analysis of DEGs between HF+THE-H and HF; (**F**) KEGG enrichment analysis of DEGs between HF and LF; (**G**) KEGG enrichment analysis of DEGs between HF+THE-H and HF; (**H**) GO enrichment analysis of Co-DEGs_214; (**I**) KEGG enrichment analysis of Co-DEGs_214.

To investigate the molecular mechanism by which THE improves HFD-induced ORCs in mice, a high-throughput sequencing approach was used to analyze the liver RNA-seq data. Approximately 70 million reads were obtained from each sample. After filtering and quality control, clean reads were mapped to the reference genome using Tophat2 [30]. Overall, 91–95% of reads were mapped to the mouse genome, and the uniformity of the mapping results indicated that the samples were reliable and comparable. Details of the mapping data are provided in Table S3. Our data suggested that the assembled results were suitable for further analysis. After assembly and annotation of the transcriptome, expression levels were calculated as FPKM based on mapped clean reads from each sample. Differential expressed gene (DEG) analysis was performed based on the sequencing data of mice liver tissues using DESeq2 package. DEGs were identified using log2FC greater than 1 and Q values less than 0.05 as screening criteria. The results are shown in Figure 5A,B. Compared to the LF group, mice on a high-fat diet had a total of 653 DEGs, with 216 genes significantly upregulated and 437 genes significantly downregulated. After intervention with high-dose THE in HFD mice, there were 362 DEGs compared to HF group mice, with 159 genes significantly upregulated and 203 genes significantly downregulated.

We further used a Venn plot to analyze the DEGs among different groups and their common changes (Figure 5C). The results showed a total of 214 common genes (Co-DEGs_214) in the DEGs of HF vs. LF and HF+THE-H vs. HF, indicating that the expression levels of these 214 genes changed significantly after HFD induction compared to the LF group. Additionally, after intervention with high-dose THE, the expression levels of these 214 DEGs tended to revert toward those of the LF group. These results indicated that high-dose THE intervention can significantly affect the liver gene expression profile of HFD mice.

Following that, GO and KEGG enrichment analyses were conducted on DEGs in each group of mice (Figure 5D–G). GO enrichment analysis of the 653 DEGs from HF vs. LF showed that lipid metabolism signaling pathways were the most altered, including fatty acid metabolic process, steroid metabolic process, long-chain fatty acid metabolic process, and lipid localization. Analysis of HF+THE-H vs. HF DEGs revealed that the ERK1 and ERK2 cascade, regulation of lipid metabolic process, and fatty acid metabolic process were the most involved pathways. The KEGG enrichment analysis results were similar to the GO enrichment results. For DEGs from HF vs. LF, the KEGG pathways were mainly enriched in steroid hormone biosynthesis, the PPAR signaling pathway, fluid shear stress, and atherosclerosis. For HF+THE-H vs. HF DEGs, the KEGG pathways were enriched in the TGF-beta signaling pathway, steroid hormone biosynthesis, phagosome, fluid shear stress, and atherosclerosis. These results indicated significant changes in lipid metabolism in the liver tissue of HFD group mice compared to the LF group. THE intervention can improve energy metabolism and lipid metabolism abnormalities induced by HFD.

To further investigate the effect of THE intervention on the liver transcriptome of mice, enrichment analysis was performed on Co-DEGs_214 from HF vs. LF and HF+THE-H vs. HF (Figure 5H,I). GO and KEGG enrichment analysis of Co-DEGs_214 also showed that lipid metabolic pathways (regulation of lipid localization, lipid localization; steroid hormone biosynthesis, fluid shear stress, and atherosclerosis) and inflammatory pathways (regulation of tumor necrosis factor superfamily cytokine production and tumor necrosis factor superfamily cytokine production; phagosome) were the most altered pathways.

### 3.5. THE Improved Glycolipid Metabolite Homeostasis in HFD-Induced Obese Mice

**Figure 6:**
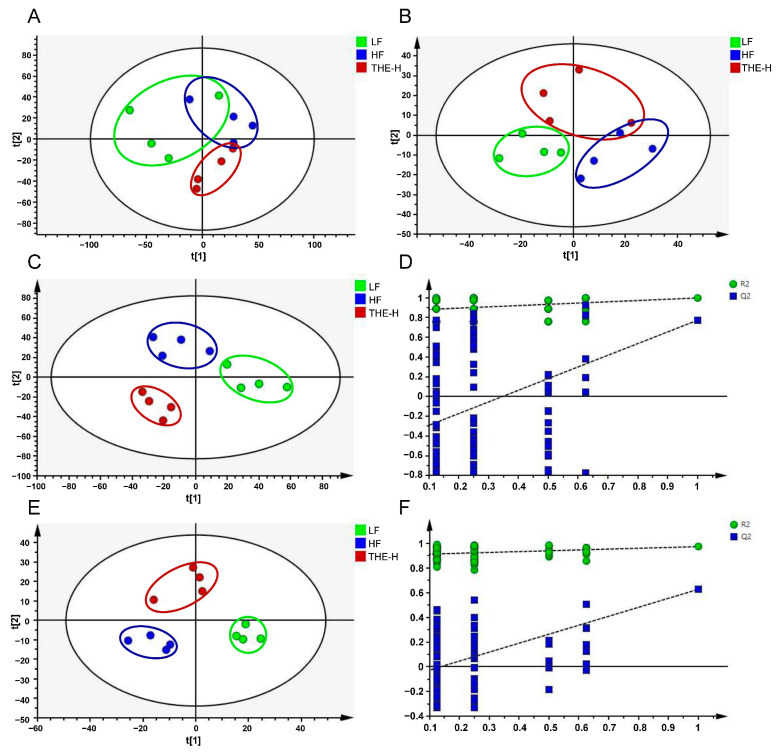
Multivariate statistical analysis of the differences in plasma metabolites in various groups of mice. ((**A**–**F**), 600 dpi resolution). (**A**) PCA-X score in positive ion mode (R^2^X = 0.661; Q^2^ = 0.1); (**B**) PCA-X score in negative ion mode (R^2^X = 0.672; Q^2^ = −0.196); (**C**) PLS-DA score in positive ion mode (R^2^X = 0.779; R^2^Y = 0.999; Q^2^ = 0.858); (**D**) PLS-DA model validation in positive ion mode (R^2^ = 0.87; Q^2^ = −0.409); (**E**) PLS-DA score in negative ion mode (R^2^X = 0.652; R^2^Y = 0.999; Q^2^ = 0.944); (**F**) PLS-DA model validation in negative ion mode (R^2^ = 0.906; Q^2^ = −0.0992).

To assess the overall changes in plasma metabolic profiles, we applied mass spectrometry-based metabolic analysis using the UHPLC-Orbitrap-MS/MS system. A typical total ion current chromatogram of plasma samples was analyzed using both positive and negative ions, which contained 4774 and 1669 metabolite ion features, respectively. First, we conducted unsupervised principal component analysis (PCA) to identify intra- and inter-class differences in metabolic profiles. Our data showed that the positive and negative ion profiles of the LF, HF, and HF+THE-H samples differed significantly among these groups (Figure 6A,B). Next, the supervised partial least squares-discriminant analysis (PLS-DA) model was applied to examine the differences in metabolites. The results further confirmed that the LF, HF, and HF+THE-H groups had distinct chemical profiles (Figure 6C,E). Additionally, the R^2^X, R^2^Y, and Q^2^ values were calculated as 0.779, 0.999, and 0.858 for positive ions, and 0.652, 0.999, and 0.944 for negative ions, respectively, suggesting that the models were successfully established without overfitting [31,32]. The permutation results were consistent (Figure 6D,F).

**Figure 7:**
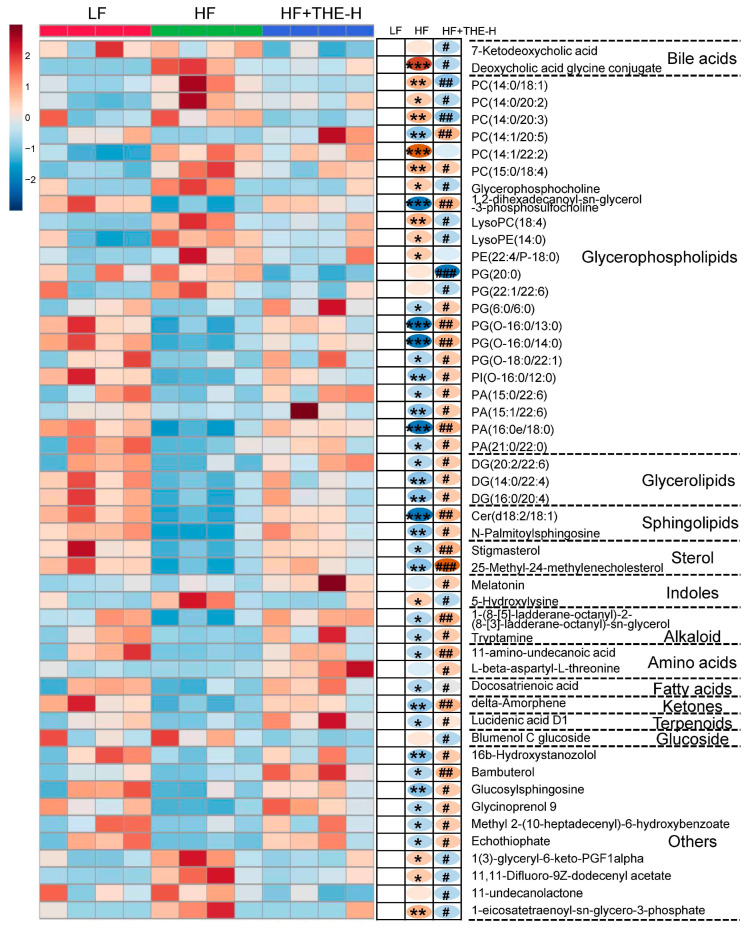
Heatmap of differential plasma metabolite contents in different groups of mice (600 dpi resolution). Boxes indicating values higher or lower than the mean are indicated in red and blue, respectively. * *p* < 0.05, ** *p* < 0.01, *** *p* < 0.001 versus LF group; # *p* < 0.05, ## *p* < 0.01, ### *p* < 0.001 versus HF group.

Then, variables with VIP > 1, *p* < 0.05, and fold change > 5 were selected as potential differential metabolites. These metabolites were identified by comparing the retention time (RT) and *m*/*z* value with published data in the literature or databases. A total of 51 metabolites were tentatively identified, including 2 bile acids, 8 phosphatidylcholine, 2 hemolytic phospholipids, 1 phosphatidylethanolamine, 6 phosphatidylglycerols, 1 phosphatidylinositol, 4 phosphatidic acids, 3 diacylglycerols, 2 sphingolipids, 2 sterols, 2 indoles, 2 alkaloids, 2 amino acids, 1 free fatty acid, 1 ketone, 1 terpene, 1 glycoside, and 10 other metabolites summarized in Table S4. The heatmap (Figure 7) showing the 51 differentially abundant metabolites provides a comprehensive overview of the metabolite contents among various groups.

The results showed that the abundances of 1 bile acid (deoxycholic acid glycine conjugate), 5 phosphatidylcholines (PC (14:0/18:1), PC (14:0/20:2), PC (14:0/20:3), PC (15:0/18:4), glycerophosphocholine), 2 lysophospholipid (lysoPC (18:4), lysoPE (14:0)), 1 indole (5-hydroxylysine), and 3 other metabolites (1(3)-glyceryl-6-keto-PGF1alpha, 11,11-difluoro-9Z-dodecenyl acetate, 1-eicosatetraenoyl-sn-glycero-3-phosphate) were significantly increased in the HF group compared to the LF group. However, the abundances of these metabolites significantly decreased after THE intervention. In addition, compared to the LF group, the abundances of 2 phosphatidylcholines (PC (14:1/20:5), 1,2-dihexadecanoyl-sn-glycero-3-phosphosulfo-choline), 4 phosphatidylglycerols (PG (6:0/6:0), PG(O-16:0/13:0), PG(O-16:0/14:0), PG(O-18:0/22:1)), 1 phosphatidylinositol (PI(O-16:0/12:0)), 4 phosphatidic acids (PA (15:0/22:6), PA (15:1/22:6), PA (16:0e/18:0), PA (21:0/22:0)), 3 diacylglycerols (DG (20:2/22:6), DG (14:0/22:4), DG (16:0/20:4)), 2 sphingolipids (Cer (d18:2/18:1), N-palmitoylsphingosine), 2 sterols (stigmasterol, 25-methyl-24-methylenecholesterol), 2 alkaloids (1-(8-[5]-ladderane-octanyl)-2-(8-[3]-ladderane-octanyl)-sn-glycerol, tryptamine), 1 amino acid (11-amino-undecanoic acid), 1 free fatty acid (docosatrienoic acid), 1 ketone (delta-amorphene), 1 terpenoid (lucidenic acid D1), and 6 other metabolites (16b-hydroxystanozolol, bambuterol, glucosylsphingosine, glycinoprenol 9, methyl 2-(10-heptadecenyl)-6-hydroxybenzoate, echothiophate) in the HF group were significantly reduced, while their abundances were significantly increased after THE intervention. In conclusion, THE intervention improved glycolipid metabolite balance in HFD-induced obese mice.

### 3.6. Correlation Analysis between Physiological Indicators Related to ORCs and Plasma Differential Metabolites

**Figure 8:**
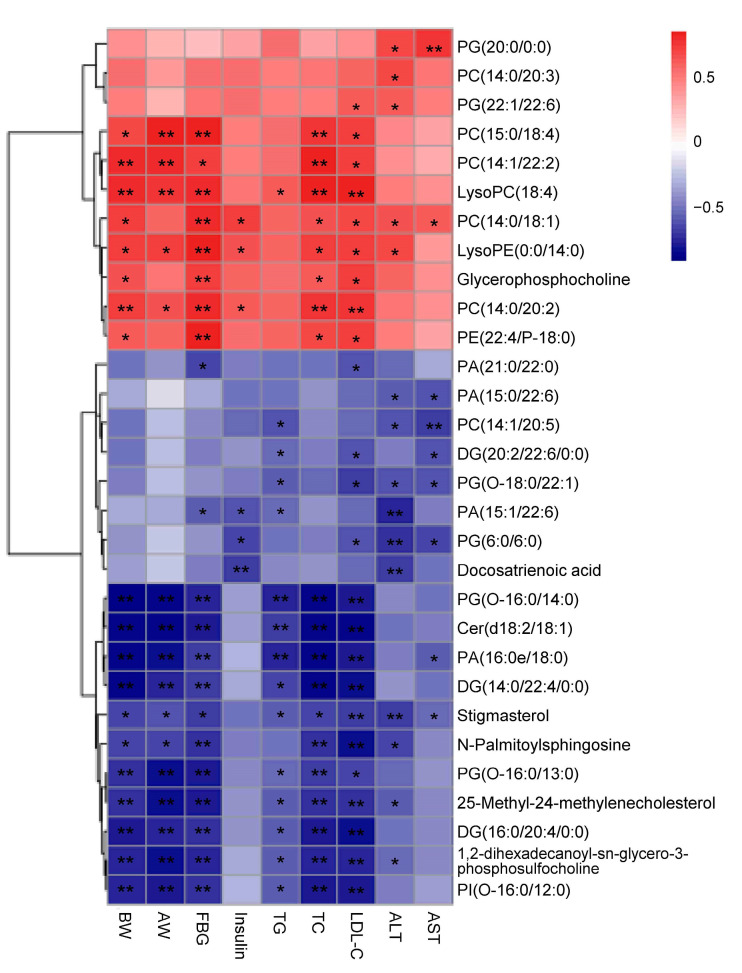
Heatmap of Spearman’s correlations between lipid differential metabolites in plasma samples and obesity-related indexes (600 dpi resolution). Notes: red boxes represent positive correlations with the phenotype of obesity complications, while blue boxes represent negative connections with the phenotype of obesity complications. BW, body weight; AW, abdominal adipose weight; FBG, fasting blood glucose. Significant correlations are marked as * *p* < 0.05, ** *p* < 0.01. Values are the mean ± SEM (*n* = 4).

**Figure 9:**
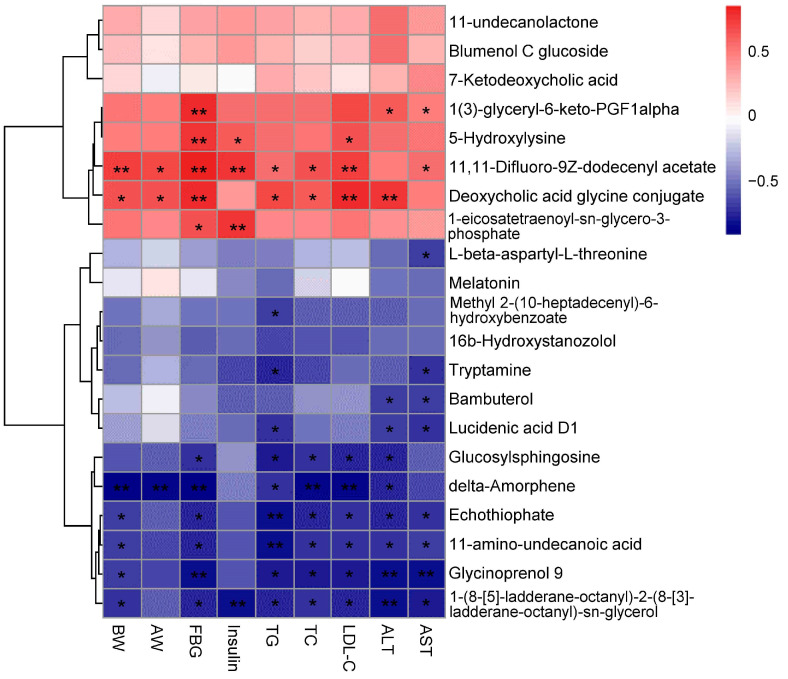
Heatmap of Spearman’s correlations between non-lipid differential metabolites in plasma samples and obesity-related indexes (600 dpi resolution). Significant correlations are marked as * *p* < 0.05, ** *p* < 0.01. Values are the mean ± SEM (*n* = 4).

According to the correlation analysis, we found that among the 51 differential metabolites screened, 47 metabolites were significantly correlated with at least one physiological indicator associated with ORCs, including body weight, abdominal adipose weight, plasma TG, plasma TC, plasma LDL-C, AST, and ALT. We first analyzed the correlations between 30 lipid differential metabolites and physiological indicators related to ORCs (Figure 8) and found that 11 lipid differential metabolites, including PG (20:0), PC (14:0/20:3), PG (22:1/22:6), PC (15:0/18:4), PC (14:1/22:2), lysoPC (18:4), PC (14:0/18:1), lysoPE (14:0), glycerophosphocholine, PC (14:0/20:2), and PE (22:4/P-18:0), were positively correlated with physiological indicators related to ORCs. Among these, PC (15:0/18:4), PC (14:1/22:2), lysoPC (18:4), PC (14:0/18:1), lysoPE (0:0/14:0), glycerophosphocholine, PC (14:0/20:2), and PE (22:4/P-18:0) were significantly positively correlated with body weight, fasting blood glucose, TC, and LDL-C. The other 19 lipid differential metabolites were negatively correlated with physiological indicators related to ORCs. Among them, PG (O-16:0/14:0), Cer(d18:2/18:1), PA (16:0e/18:0), DG (14:0/22:4), stigmasterol, PG (O-16:0/13:0), 25-methyl-24-methylecholesterol, DG (16:0/20:4), 1,2-dihexadecanoyl-sn-glycero-3-phospholulfocaline, and PI (O-16:0/12:0) were strongly negatively correlated with body weight, abdominal fat weight, fasting blood glucose, TG, TC, and LDL-C. Next, we analyzed 21 screened non-lipid differential metabolites and their correlations with physiological indicators related to ORCs (Figure 9). The results indicatedthat 11,11-difluoro-9Z decodenyl acetate and deoxycholic acid glycine conjugate were significantly positively correlated with body weight, abdominal fat weight, fasting blood glucose, TG, TC, and LDL-C. Conversely, delta-amorphene, echothiophate, 11-amino undecanoic acid, glycinoprenol 9, and 1-(8-[5]-ladderane-octanyl)-2-(8-[3]-lactaterone octyl)-sn-glycerol were strongly negatively correlated with body weight, fasting blood glucose, TG, TC, LDL-C, and ALT.

## 4. Discussion

ORCs are a global public health concern, generally characterized by fat accumulation, dyslipidemia, oxidative stress, and systemic low-grade inflammation [33]. Various natural plant-based substances have been identified as promising and feasible alternatives for preventing and alleviating ORCs [25,34,35]. As an important component of tea, THE supports neuroprotection and cognitive function and has been shown to impact the body’s nutritional metabolism [19,20]. However, the potential effects and mechanisms of THE as a food additive for reducing ORCs remain unclear. The present study examined this subject and found that THE significantly relieved ORCs induced by HFD, including decreasing body weight gain and fat deposition, improving glycolipid metabolism disorders, and relieving inflammation, although food and energy intakes in mice did not decrease accordingly (Figure S1). This indicates that THE’s improvement of ORCs is not dependent on the restriction of energy intake. Moreover, compared with low-dose THE, high-dose THE showed a more significant improvement effect on ORCs. 

A common feature of obesity is lipid accumulation in the liver, which can result in oxidative stress in liver cells, leading to liver injury, fibrosis, and even cirrhosis, as well as other complications [36,37,38]. THE has been previously reported to provide liver protection. Liang et al. reported that THE inhibits the progression of hepatic steatosis in non-alcoholics by modulating hepatocyte lipid metabolism [39]. In our study, THE treatment suppressed not only lipid accumulation in the liver of mice on HFD, but also ALT and AST activities, thereby alleviating liver injury in mice fed HFD. According to these results, THE could prevent liver damage and alleviate nonalcoholic fatty liver disease. Moreover, we found that the improvement effect in the high-dose THE group was substantially greater than that in the low-dose THE group.

Following this, we explored the molecular mechanism underlying THE amelioration of ORCs induced by HFD. Sterol regulatory element-binding protein 1 (SREBP1), acetyl-CoA carboxylase (ACC), fatty acid synthase (FAS), and stearoyl-CoA desaturase 1 (SCD1) are critical enzymes for fatty acid synthesis. Studies have shown that green tea lowers blood glucose levels and improves blood lipid parameters by regulating the expression of FAS, ACC, and SREBP-1 [40,41]. Our results indicated that mRNA expression of *Srebp-1*, *Acc*, *Fasn,* and *Scd1* was suppressed by THE intervention in the liver of mice (Figure 3). Interestingly, intervention with high-dose THE exhibited a more profound suppression effect. The RNA-seq data from liver tissues also showed that the expression levels of lipogenesis-related genes *Lxrα*, *Srebf1*, *Pparγ*, *Accα*, *Fasn*, and *Scd1* were all decreased by high-dose THE intervention (Figure S2). Our study demonstrated that high-dose THE effectively suppressed fatty acid synthesis in the liver of HFD mice. Lin et al. reported that THE downregulated the expression of FAS, SREBP1, and HMGCR via insulin and AMP-activated protein kinase signaling pathways [21]. These results align closely with our findings.

The inflammatory response plays a crucial role in ORC development [42,43]. A variety of inflammatory cytokines contributes to the development of diabetes and cardiovascular diseases caused by ORCs [44]. It has been demonstrated in previous studies that THE may prevent carbon tetrachloride-induced liver fibrosis by inhibiting nuclear factor κB and inhibiting transforming growth factor (TGF) [45]. Wang et al. found that THE potentially ameliorates LPS-induced inflammation and acute liver injury through inactivation of the NF-κB signaling pathway [46]. Our results revealed that both low-dose and high-dose THE significantly decreased the expression of *TNFα*, *IL-6,* and *MCP-1* genes. However, only high-dose THE significantly reduced *IL-1β* expression compared to HF group mice. This indicates that THE exhibited a dose-dependent effect to inhibit *IL-1β* expression (Figure 4). Previous research has shown that IL-1β plays an pivotal role in the activation and regulation of NLRP3 inflammasome. Production of IL-1β via NLRP3 can contribute to the pathogenesis of inflammatory disease, whereas aberrant IL-1β secretion through inherited NLRP3 mutations causes autoinflammatory disorders [47]. And the RNA-seq data from liver tissues also showed that inflammatory related genes *Tnfsf12*, *Tnfsf13*, *IL-4*, *IL-15*, *IL-16*, and *IL-33* were all downregulated by high-dose THE treatment (Figure S3).

Previous research reported that THE improves fatty liver-related pathological processes, such as lipid deposition, insulin resistance, inflammatory, and fibrosis [39,45,46]. Our RNA-seq data from liver tissues showed that treatment with high-dose THE significantly regulated many genes induced by HFD (Figure 5). There were 214 DEGs in common between HF vs. LF and HF+THE-H vs. HF.GO and KEGG pathway analysis of the 214 DEGs revealed that lipid metabolic pathways (regulation of lipid localization, lipid localization; steroid hormone biosynthesis, fluid shear stress, and atherosclerosis) and inflammatory pathways (regulation of tumor necrosis factor superfamily cytokine production and tumor necrosis factor superfamily cytokine production; phagosome) were the most modulated pathways in response to THE intervention. 

Plasma metabolites are crucial markers of the systemic metabolic profile, reflecting the overall health of the host [28]. Obesity is often accompanied by metabolism disorders of plasma metabolites [48]. This study examined the overall changes in plasma metabolic profiles after high-dose THE supplementation using mass spectrometry-based metabolomic approaches. We screened and identified a total of 51 differential metabolites, including 30 lipid-related and 21 non-lipid-related differential metabolites. Pathway analysis revealed that five lipid metabolism pathways were affected by THE, including glycerophospholipid metabolism, tryptophan metabolism, pentose and glucuronic acid interconversion metabolism, glycolysis and glucose production metabolism, and linoleic acid metabolism (Figure S4). 

The 30 differential lipid metabolites that were significantly altered by THE supplementation included 20 glycerophospholipids (7 phosphatidylcholines, 2 lyso-phospholipids, 6 phosphatidylglycerols, 1 phosphatidylinositol, and 4 phosphatidic acids), 3 diacylglycerols, 2 sphingolipids, 2 sterols, and 1 free fatty acid. Studies have shown that glycerophospholipids play important structural and functional roles in cell membranes, such as participating in the recognition and transmission of intercellular signals [49]. In this study, THE supplementation decreased the levels of PC(14:0/18:1), PC(14:0/20:2), PC(14:0/20:3), PC (15:0/18:4), lysoPC (18:4), glycerophosphocholine, lysoPE (14:0), PG (20:0), and PG (22:1/22:6) and increased the levels of PC (14:1/20:5), 1,2-dihexadecanoyl-sn-glycero-3-phosphosulfocholine, PG(6:0/6:0), PG(O-16:0/13:0), PG(O-16:0/14:0), PG(O-18:0/22:1), PI(O-16:0/12:0), PA (15:0/22:6), PA (15:1/22:6), PA (16:0e/18:0), and PA (21:0/22:0) in the plasma of HFD-induced obese mice. 

Phosphatidylcholine (PC) and phosphatidylethanolamine (PE) are important surface components of plasma lipoproteins [50], including very low-density lipoprotein (VLDL), low-density lipoprotein (LDL), and high-density lipoprotein (HDL). The relative levels of PC and PE in the plasma of HFD-induced obese mice were reduced after intervention with THE, suggesting that THE has anti-dyslipidemia effects. Lysophosphatidylcholine (lysoPC) in plasma has been significantly associated with the occurrence of atherosclerosis [51]. The correlation analysis showed that lysoPC (18:4) was significantly positively correlated with ORC-related indicators. The decrease in the relative content of lysoPC (18:4) in the THE intervention group also indicated the preventive and protective effects of THE on atherosclerosis. 

Additionally, phosphatidic acid (PA) is an important phospholipid signaling molecule involved in intracellular and extracellular signal transduction. Increasing evidence suggests that the intermediate products of phospholipid metabolism, such as PA and lysophosphatidic acid (LPA), are important second messengers that play roles in various cellular functions [52]. After intervention with THE, the levels of four phospholipid metabolites, PA (15:0/22:6), PA (15:1/22:6), PA (16:0e/18:0), and PA (21:0/22:0), were significantly increased in the plasma of HFD-induced obese mice. Among these, PA (16:0e/18:0) was strongly negatively correlated with ORC-related indicators.

Fatty liver is characterized by high levels of glycerolipids [53], and an elevated level of monounsaturated DGs in the liver is associated with insulin resistance [54]. However, Wu et al. reported that after EGCG intervention, levels of DGs, including DG (16:0/18:1), DG (18:0/18:1), and DG (18:1/18:1), were not increased in the liver of Lepr KO rats. Instead, DG species that contained polyunsaturated fatty-acyl chains and high levels of acyl-chain carbons (>38 carbons) were selectively increased in Lepr KO rats [55]. Our study found that THE intervention also selectively increased the levels of certain DG species with polyunsaturated fatty-acyl chains and high numbers of acyl-chain carbons in HFD-induced obese mice. Due to the influence of lipid structure, unsaturation level, and carbon chain length on biological functions, it is understandable that different types of DGs exhibit different expression trends.

Sphingolipids are also an important component of the plasma membrane, playing a crucial role in cellular interactions and recognition [56]. Multiple studies have shown that sphingolipids are a key metabolite linking obesity with T2D, cardiovascular disease, and metabolic disorders [57,58]. The correlation analysis showed that Cer (d18:2/18:1) and N-palmitoylsphingosine were significantly negatively correlated with ORC-related indicators. We found that THE intervention restored the relative levels of Cer (d18:2/18:1) and N-palmitoylsphingosine in the plasma of HFD group mice. 

Stigmasterol and 24-methyl-25-methylcholesterol are plant sterols with structures similar to cholesterol [59]. Stigmasterol has been reported to have strong pharmacological effects, such as anticancer, anti-osteoarthritis, anti-inflammatory, anti-diabetes, immune regulation, antibacterial, and antioxidant properties [60]. We found that THE also had a beneficial effect by increasing the content of stigmasterol in the plasma of HFD-induced obese mice.

Among the 21 non-lipid differential metabolites, THE supplementation resulted in decreased levels of 2 bile acids (7-ketodeoxycholic acid, deoxycholic acid glycine conjugate), 1 indole (5-hydroxylysine), 1 glycoside (blumenol C glucoside), and 4 other metabolites (1(3)-glyceryl-6-keto-PGF1alpha, 11,11-difluoro-9Z-dodecenyl acetate, 11-undecanolactone, 1-eicosatetraenoyl-sn-glycero-3-phosphate). Conversely, it increased the levels of 2 amino acids (11-amino-undecanoic acid, L-beta-aspartyl-L-threonine), 1 free fatty acid (docosatrienoic acid), 1 ketone (delta-amorphene), 1 terpenoid (lucidenic acid D1), and 6 other metabolites (16b-hydroxystanozolol, bambuterol, glucosylsphingosine, glycinoprenol 9, methyl 2-(10-heptadecenyl)-6-hydroxybenzoate, echothiophate).

Studies have shown that bile acids are synthesized from cholesterol in the liver and have multiple physiological functions, including maintaining cholesterol balance and aiding lipid absorption [61]. The hydrophilicity or hydrophobicity of bile acids is an important factor determining their biological activity, such as their ability to affect cell membrane shuttling, FXR binding capacity, and intestinal absorption of dietary cholesterol and lipids [60]. Increased levels of hydrophobic bile acids may disrupt the blood glucose balance by stimulating inflammation and causing endoplasmic reticulum stress [62]. Deoxycholic acid glycine conjugate is a typical hydrophobic bile acid. Many studies have shown that elevated glycodeoxycholic acid levels are closely related to insulin resistance in diabetes [63]. In our study, the glycodeoxycholic acid level was significantly increased in the plasma of high-fat diet-induced obese mice. Correlation analysis revealed that glycodeoxycholic acid was significantly positively correlated with ORC-related indicators, while THE intervention significantly reduced the content of glycodeoxycholic acid in the plasma of HFD mice, indicating that the effect of THE improving ORCs may be related to the improvement of bile acid metabolism. 

Melatonin is a hormone produced by the pineal gland in the brain that exhibits antioxidant, anti-inflammatory, and anticancer properties [63,64] and is involved in regulating various physiological processes, such as aging and tissue and organ development [65,66,67,68]. Wang et al. found that THE enhances the circadian rhythm of vascular smooth muscle, and supplementing with THE significantly improved the amplitude of rhythm gene expression in aged mice. The increase in melatonin content in mouse plasma further supports our findings [26]. In this study, THE significantly increased the relative content of melatonin in the plasma of high-fat diet-induced obese mice, explaining the ameliorative effects of THE on ORCs.

Previous studies have proved that THE is a safe food addictive at high doses of 4000 mg/kg body weight doses in the diet for 13 weeks in rats [69]. Additionally, no dietetic exposure limits have been suggested for THE by the Japan Food Additives Association [20]. Kahathuduwa et al. used a high dosage of L-theanine (2.5 mg/kg) plus caffeine (2.0 mg/kg) to treat boys (8–15 years) with attention deficit hyperactivity disorder (ADHD). They concluded that the L-theanine–caffeine combination may be a potential therapeutic option for ADHD-associated impairments in sustained attention, inhibitory control, and overall cognitive performance. And no side effects were reported [70]. In this study, our results also indicated that 900 mg/kg of THE significantly improved ORCs, and no side effects were observed in mice. A daily dosage of 900 mg/kg of THE supplemented to mice is equivalent to 5.12 g of THE consumed by an adult human (70 kg). Our data showed that a dose of 400 mg/kg of theanine significantly alleviated ORCs. Previous research indicated that a dose of 400 mg/kg of theanine can already have a positive impact on depression, improving cognition and sleep [71,72]. The high-dose THE used in this study is a relatively high concentration. Thus, attention should be paid when using this high concentration of THE in clinical trials. And further research is needed to determine the effects of high-dose THE on the nervous system in the future. In spite of that, our research still provides a new perspective on THE’s potential applications in the treatment of ORCs.

## 5. Conclusions

In summary, our study suggests that high-dose THE can effectively ameliorate ORCs by reducing body weight gain and fat deposition, improving glycolipid metabolism disorders, suppressing inflammation, and alleviating hepatic steatosis induced by HFD in mice. These effects of THE are linked to the activation of lipid catabolic pathways and suppression of inflammatory pathways, which inhibit hepatic steatosis and inflammation in the liver of HFD-fed mice. Furthermore, THE positively maintained the plasma metabolites balance of PC(14:0/18:1), lysoPE(14:0), PA(16:0e/18:0), stigmasterol, and deoxycholic acid glycine conjugate and regulated the glycerophospholipid metabolism pathway and tryptophan metabolism pathway. Therefore, high-dose THE may modulate host metabolism to relieve inflammation and dyslipidemia, thereby alleviating ORCs. This study provides theoretical support for the potential use of THE as a food additive with properties that alleviate ORCs. However, the exact molecular mechanism of how THE alleviates ORCs needs to be further investigated. In addition, the effects of high doses of THE on the nervous system will require further research in the future.

## Data Availability

The original contributions presented in the study are included in the article and supplementary material, further inquiries can be directed to the corresponding author.

## Supplementary Materials

The following supporting information can be downloaded at: https://www.mdpi.com/article/10.3390/foods13182977/s1, Text S1: Plasma metabolomics analysis method; Figure S1: The food intake and water intake in different group mice; Figure S2: The hepatic lipogenesis-related gene expression was quantified by FPKM in the liver tissues of various groups of mice; Figure S3: The inflammatory response gene expression was quantified by FPKM in the liver tissues of various groups of mice; Figure S4: Metabolic pathway analysis of differential plasma metabolites in various groups of mice; Table S1: The composition of the experimental diet; Table S2: Real-time PCR primers used in this study; Table S3: Overview of mapping of RNA-seq reads against the reference genome; Table S4: Differential plasma metabolites in different groups of mice by metabolomics analysis based on UHPLC-Orbitrap-MS/MS.
