# Supplementary material for: L-Theanine Ameliorates Obesity-Related Complications Induced by High-Fat Diet in Mice: Insights from Transcriptomics and Metabolomics
Source: Foods. 2024 Sep 19;13(18):2977. doi: 10.3390/foods13182977 (PMC11431230; doi:10.3390/foods13182977)
Supplement: Supplementary file 1 [file foods-13-02977-s001.zip › foods-3173257-supplementary.pdf]

Supplementary Materials

Text S1. Method for plasma metabolomics analysis

Plasma samples were extracted as follows: 140 μL acetonitrile (including 5 μg/mL glibenclamide as an internal standard) was added to 20 μL of plasma and vortexed for 3 min. The mixture was then incubated in an ice bath for 20 minutes before being centrifuged at 16000 rpm and 4°C for 10 minutes to collect supernatant. Subsequently, the supernatant was filtered through a 0.22 μm organic filter membrane, and the filtrate was used for metabolomics analysis. Metabolite analysis was performed using an Ultimate HPLC system and orbitrap mass spectrometry (UHPLC-Orbitrap-MS/MS) (Thermo Fisher Scientific, MA, USA). Each plasma sample was analyzed in four parallel experiments

Sample chromatographic analysis was performed using the Ultimate HPLC system (Thermo Fisher Scientific, USA). Sample separation was conducted using a Thermo Hypersil Gold column (Thermo Fisher Scientific, USA) in both positive and negative ion modes. The mobile phase A for positive ion mode was 0.1% formic acid in water (mobile phase A), and acetonitrile (mobile phase B). For negative ion mode, the mobile phase A consisted of 5 mM ammonium acetate in water and pure acetonitrile as mobile phase B. The flow rate for both modes was 0.35 mL/min, with the column temperature set at 40 ℃, the automatic sampler temperature at 15 ℃, and an injection volume of 5 μ L. The gradient elution was as follows: 2% B from 0-1 min; 2-98% B from 1-9 min; 98% B from 9-12 min; 98-2% B form 12-12.1 min; and 2% B from 12.1-15 min.

Mass spectrometry detection was carried out using the Q Empirical Focus Oribitrap MS/MS system (Thermo Fisher Scientific, USA) equipped with heated electrospray ionization (HESI) source. The spray voltage was set to 3.5 kV and 2.8 kV for positive and negative ionization modes, respectively. The mass range was 150-1200 m/z; with the capillary temperature maintained between 320-330℃. The sheath gases (N2) and auxiliary gas (N2) were 50 and 10 psi, respectively.

Raw data were acquired and processed using Progenesis QI （Waters Corporation, Milford, USA）, which included molecular feature extraction, data alignment, data filtering, handling of missing values processing, and data normalization. The processed data were then imported into SIMCA 14.1 for principal component analysis (PCA) and orthogonal projections to latent structure discriminant analysis (PLS-DA). The variable importance in projection (VIP) value was used to assess the contribution of variables to the differences between groups. Criteria for screening differential metabolites between groups were set at VIP > 1, $p < 0.05$ (T-test) with fold change > 5. Metabolite identification was performed by comparing the m/z, exact molecular weight, retention time, and MS/MS information with those of metabolites in the METLIN database and Human Metabolome Database (HMDB), with an accuracy error of 5 ppm. Enrichment analysis and metabolic pathway analysis of differential metabolites were conducted using MetaboAnalyst 5.0 (https://www.metaboanalyst.ca).

Table S1. The composition of the experimental diets

Ingredient (g)	low-fat diet	high-fat diet
Casein	191	262
Cornstarch	484	0
Dextrin	120	164
Sucrose	66	90
Soybean oil	24	33
Lard	19	321
Cellulose	48	65
Mineral mix	33	46
Vitamin mix	10	13
L-Cystine	3	4
Line bitartrate	2	3
TBHQ	0.01	0.07
Total	1000	1000
Total energy		
Protein, %	20.6	19
Fat, %	12.0	60
Carbohydrate, %	67.4	21
Energy, Kcal/g	3.616	5.2

Table S2. Real-time PCR primers used in this study

Gene	Primer sequences Forward (5′-3′)	Primer sequences Reverse (5′-3′)
Srebf1	GATCCAGCCTTTGAGGATAGCC	CCGTAGCATCAGAGGGAGTGAG
Accα	AGGAGGGAAAGGGATCAGAAAAG	CAGAGCAGTCACGACCAAACAAA
Fasn	CGTGTGACCGCCATCTATATCG	TGAGGTTGCTGTCGTCTGCAGTCTT
Scd1	TCCTCCTTGGATTGTGTAGAAACTT	AATGTCAGAAGAAATCAGGTGGGTA
TNFα	GGCTGCCCCGACTACGT	ACTTTCTCCTGGTATGAGATAGCAAAT
IL-1β	TCGCTCAGGGTCACAAGAAA	CATCAGAGGCAAGGAGGAAAAC
IL-6	CTGCAAGAGACTTCCATCCAGTT	AGGGAAGGCCGTGGTTGT
MCP-1	CTTCCTCCACCACCATGCA	CCAGCCGGCAACTGTGA
18S	GGGTCGGGAGTGGGTAATTT	AGAAACGGCCACATCCAA

Table S3. Overview of mapping of RNA-seq reads against the reference genome

Sample Name	Total Clean Reads	Total Clean Bases	%GC	Clean Reads Q30 (%)	Mapped Reads	Mapped Reads Ratio (%)
LF1	73,146,433	21,890,893,814	49.50	95.00	68,345,590	93.44
LF2	74,857,712	22,407,223,312	49.50	95.10	70,669,050	94.40
LF3	87,642,336	26,219,261,708	49.09	95.13	80,421,746	91.76
HF1	81,766,665	24,435,743,960	49.76	95.10	76,166,023	93.15
HF2	79,566,639	23,809,534,632	50.17	95.11	75,468,445	94.85
HF3	78,223,752	23,418,083,626	50.24	95.03	74,649,594	95.43
HF+THE-H1	73,654,906	22,056,700,174	50.23	95.08	69,590,658	94.48
HF+THE-H2	75,492,138	22,587,210,930	49.38	95.14	69,798,989	92.46
HF+THE-H3	81,735,019	24,463,113,756	49.39	95.07	75,778,971	92.71

Table S4. Differential plasma metabolites in different group of mice by metabolomics analysis based on UHPLC-Orbitrap-MS/MS

Number	Description	Chemical Class	Compound ID	Ion	Retention time (min)	Adducts	m/z	Formula	FC (LF/HF)	FC (HF+THE-H/HF)
1	7-Ketodeoxycholic acid	Bile acids	HMDB0000391	pos (+)	3.3339	M+NH4	424.3057	C24H38O5	0.926095	0.135891
2	Deoxycholic acid glycine conjugate	Bile acids	HMDB0000631	pos (+)	10.36977	M+K	488.2773	C26H43NO5	0.01511	0.40138
3	PC (14:0/18:1)	PCs	HMDB0007872	neg (-)	9.60185	M+FA-H	776.5435	C40H78NO8P	0.097705	0.145852
4	PC (14:0/20:2)	PCs	HMDB0007880	neg (-)	9.932183	M+FA-H	802.5591	C42H80NO8P	0.150284	0.526275
5	PC (14:0/20:3)	PCs	HMDB0007881	neg (-)	10.93553	M+FA-H	800.5433	C42H78NO8P	0.148326	0.269025
6	PC (14:1/20:5)	PCs	HMDB0007918	neg (-)	8.5346	M-H	748.4939	C42H72NO8P	16.36043	27.42652
7	PC (14:1/22:2)	PCs	HMDB0007921	neg (-)	9.7311	M+FA-H	828.5736	C44H82NO8P	0.173564	0.849564
8	PC (15:0/18:4)	PCs	HMDB0007943	neg (-)	7.382883	M+FA-H	784.5121	C41H74NO8P	0.149537	0.692733
9	Glycerophosphocholine	PCs	HMDB0000086	pos (+)	0.85895	M+Na	280.0921	C8H20NO6P	0.135633	0.321075
10	1,2-dihexadecanoyl-sn-glycero-3-phosphosulfocholine	PCs	LMGP00000050	pos (+)	19.99143	M+H-H2O	719.5041	C39H77O8PS	5.756121	4.312538
11	LysoPC (18:4)	LPCs	HMDB0010389	pos (+)	11.15853	M+H	516.3087	C26H46NO7P	0.032501	0.523929
12	LysoPE (14:0)	LPEs	HMDB0011470	pos (+)	16.3492	2M+NH4	868.5432	C19H40NO7P	0.171501	0.648698
13	PE (22:4/P-18:0)	PEs	HMDB0009610	neg (-)	9.431967	M+FA-H	824.5787	C45H82NO7P	0.163307	0.958176
14	PG (20:0)	PGs	LMGP04050015	pos (+)	3.937333	M+H	541.3484	C26H53O9P	0.664446	0.089257
15	PG (22:1/22:6)	PGs	LMGP04010755	pos (+)	15.88852	M+K	915.5535	C50H85O10P	0.117389	0.32513
16	PG (6:0/6:0)	PGs	LMGP04010030	pos (+)	15.64288	M+NH4	460.2305	C18H35O10P	4.844887	9.629509
17	PG(O-16:0/13:0)	PGs	LMGP04020002	pos (+)	17.51703	M+H	667.4907	C35H71O9P	5.229663	3.75062
18	PG(O-16:0/14:0)	PGs	LMGP04020003	pos (+)	18.70853	M+H	681.5061	C36H73O9P	7.729985	5.769739
19	PG(O-18:0/22:1)	PGs	LMGP04020038	pos (+)	15.87515	M+NH4	836.6754	C46H91O9P	7.622788	8.598548
20	PI(O-16:0/12:0)	PIs	LMGP06020002	pos (+)	16.90753	M+Na	763.4725	C37H73O12P	6.353518	4.028986
21	PA (15:0/22:6)	PAs	LMGP10010158	neg (-)	11.6977	M-H	705.4513	C40H67O8P	5.5595	5.302814

Table S4. (Continued)

Number	Description	Chemical Class	Compound ID	Ion	Retention time (min)	Adducts	m/z	Formula	FC (LF/HF)	FC (HF+THE-H/HF)
22	PA (15:1/22:6)	PAs	LMGP10010189	pos (+)	18.34653	M+H-H2O	687.4384	C40H65O8P	5.163296	3.205454
23	PA (16:0e/18:0)	PAs	HMDB0011145	pos (+)	18.97853	M+H-H2O	645.5215	C37H75O7P	5.254436	5.084064
24	PA (21:0/22:0)	PAs	LMGP10010697	pos (+)	17.19478	M+NH4	820.6805	C46H91O8P	5.486748	3.204113
25	DG (20:2/22:6)	DGs	HMDB0007440	neg (-)	15.89912	M-H	691.5292	C45H72O5	6.585197	5.4146
26	DG (14:0/22:4)	DGs	HMDB0007031	pos (+)	18.77188	M+Na	639.4957	C39H68O5	7.329877	4.823923
27	DG (16:0/20:4)	DGs	HMDB0007113	pos (+)	18.5159	M+H-H2O, M+Na	639.4955	C39H68O5	5.376174	3.559155
28	Cer (d18:2/18:1)	Sphingolipids	LMSP02010025	pos (+)	19.37802	M+Na	584.5013	C36H67NO3	6.937354	4.778835
29	N-Palmitoylsphingosine	Sphingolipids	HMDB0000790	pos (+)	18.12303	M+H	538.5195	C34H67NO3	5.864055	5.150684
30	Stigmasterol	Sterol	HMDB0000937	pos (+)	15.4245	M+H-H2O	395.3671	C29H48O	6.517504	5.087918
31	25-Methyl-24-methylenecholesterol	Sterol	HMDB0030078	pos (+)	17.44072	M+H-H2O	395.3672	C29H48O	5.725517	4.23773
32	Melatonin	Indoles	HMDB0001389	pos (+)	12.95583	2M+H	465.2498	C13H16N2O2	1.053393	5.385113
33	5-Hydroxylysine	Indoles	HMDB0000450	pos (+)	14.95558	2M+K	363.1625	C6H14N2O3	0.199673	0.379936
34	1-(8-[5]-ladderane-octanyl)-2-(8-[3]-ladderane-octanyl)-sn-glycerol	Alkaloid	LMGL02030030	neg (-)	15.82335	M-H	633.5242	C43H70O3	4.427771	5.024817
35	Tryptamine	Alkaloid	HMDB0000303	pos (+)	12.92908	2M+Na	343.1902	C10H12N2	13.32086	17.83999
36	11-amino-undecanoic acid	Amino acids	LMFA01100004	pos (+)	14.10278	2M+K	441.3085	C11H23NO2	24.52426	17.02687
37	L-beta-aspartyl-L-threonine	Amino acids	HMDB0011169	pos (+)	0.9169	M+Na, M+K	257.0744	C8H14N2O6	1.528847	11.10239
38	Docosatrienoic acid	Fatty acids	HMDB0002823	pos (+)	12.39185	M+NH4	352.3208	C22H38O2	5.119482	5.322882
39	delta-Amorphene	Ketones	HMDB0030644	pos (+)	16.40268	2M+H	409.3826	C15H24	6.695267	4.996845
40	Lucidenic acid D1	Terpenoids	HMDB0038199	pos (+)	15.3309	M+NH4	488.2621	C27H34O7	5.206415	10.95977

Table S4. (Continued)

Number	Description	Chemical Class	Compound ID	Ion	Retention time (min)	Adducts	m/z	Formula	FC (LF/HF)	FC (HF+THE/HF)
41	Blumenol C glucoside	Glucoside	HMDB0040668	pos (+)	3.6848	M+NH4	390.2486	C19H32O7	0.457334	0.063199
42	16b-Hydroxystanozolol	Others	HMDB0003166	pos (+)	17.38723	2M+Na	711.4801	C21H32N2O2	5.776438	3.659483
43	Bambuterol	Others	HMDB0015478	pos (+)	17.31147	M+NH4	385.2451	C18H29N3O5	3.277926	7.701082
44	Glucosylsphingosine	Others	HMDB0000596	pos (+)	17.90723	M+Na	484.3245	C24H47NO7	5.379838	3.73773
45	Glycinoprenol 9	Others	HMDB0038538	pos (+)	18.24783	M+Na	659.608	C45H80O	4.776584	6.647387
46	Methyl 2-(10-heptadecenyl)-6-hydroxybenzoate	Others	HMDB0038523	pos (+)	13.46618	M+NH4	406.3317	C25H40O3	6.318248	6.30658
47	Echothiophate	Others	HMDB0015190	pos (+)	12.60212	M+NH4	274.1463	C9H23NO3PS+	22.53171	19.26315
48	1(3)-glyceryl-6-keto-PGF1alpha	Others	LMFA03010187	neg (-)	11.42582	2M-H	887.5357	C23H40O8	0.131573	0.339302
49	11,11-Difluoro-9Z-dodecenyl acetate	Others	LMFA07010259	pos (+)	11.97988	2M+K	563.3129	C14H24F2O2	0.13704	0.408726
50	11-undecanolactone	Others	LMFA07040001	pos (+)	1.020217	M+NH4	202.1802	C11H20O2	0.626975	0.185863
51	1-eicosatetraenoyl-sn-glycero-3-phosphate	Others	HMDB0062312	neg (-)	9.172983	M-H2O-H	439.2251	C23H39O7P	0.03558	0.433763

Figure S1

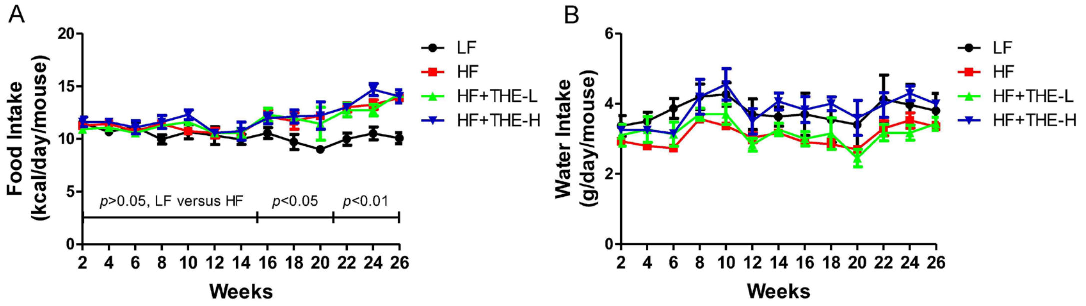

Figure S1. The food intake (A) and water consumption (B) in different groups of mice. Values are the mean ± SEM (n = 6).

Figure S2

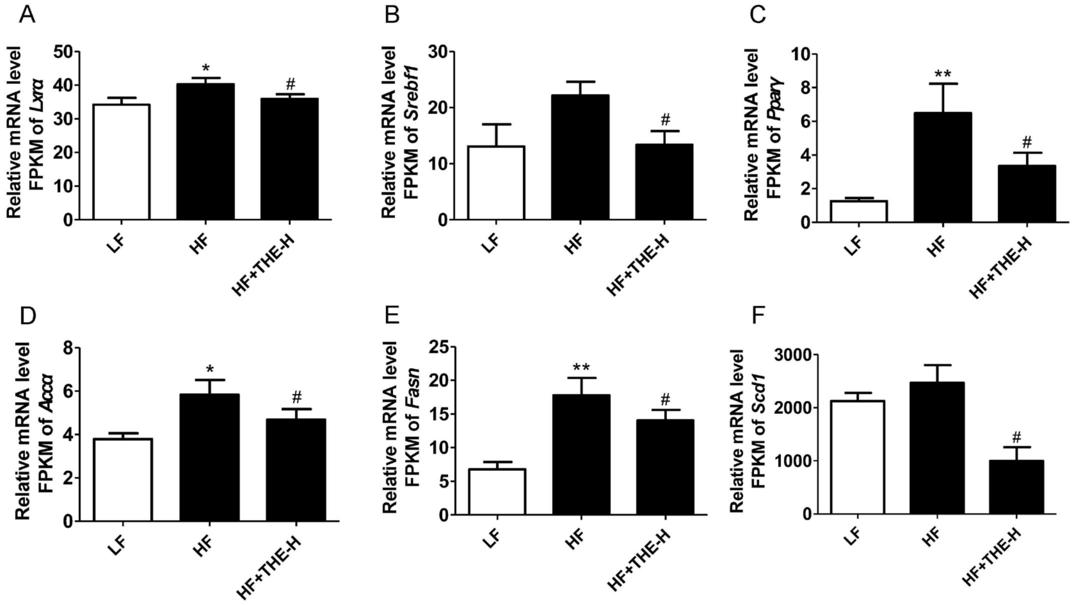

Figure S2. The hepatic lipogenesis related gene expression was quantified by FPKM in the liver tissue of various group mice. Notes: LF, LFD feeding mice; HF, HFD feeding mice; HF+THE-H, HFD with high-dose of L-theanine supplement feeding mice. The mRNA levels of *Lxrα* (A), *Srebf1* (B), *Pparγ* (C), *Accα* (D), *Fasn* (E), and *Scd1* (F) are showing. Values are means±SEM, n=3. *p < 0.05, **p < 0.01 when compared to LF; #p < 0.05, compared with HF.

Figure S3

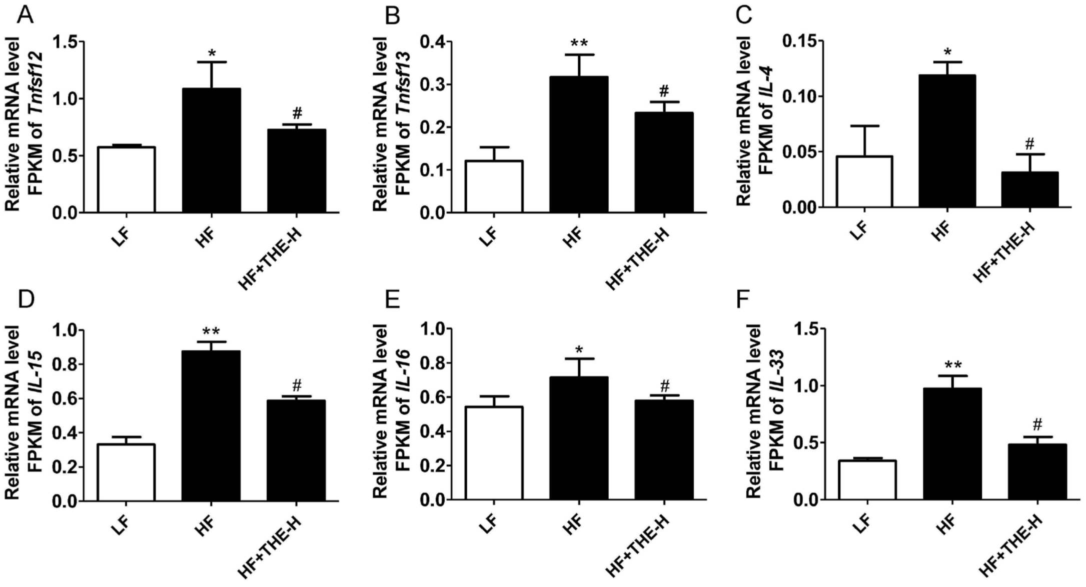

Figure S3. The inflammatory response gene expression was quantified by FPKM in the liver tissue of various group mice. The mRNA levels of *Tnfsf12* (A), *Tnfsf13* (B), *IL-4* (C), *IL-15* (D), *IL-16* (E), and *IL-33* (F) are showing. Values are means±SEM, n = 3. **p* < 0.05, ***p* < 0.01 when compared to LF, #*p* < 0.05 compared with HF.

Figure S4

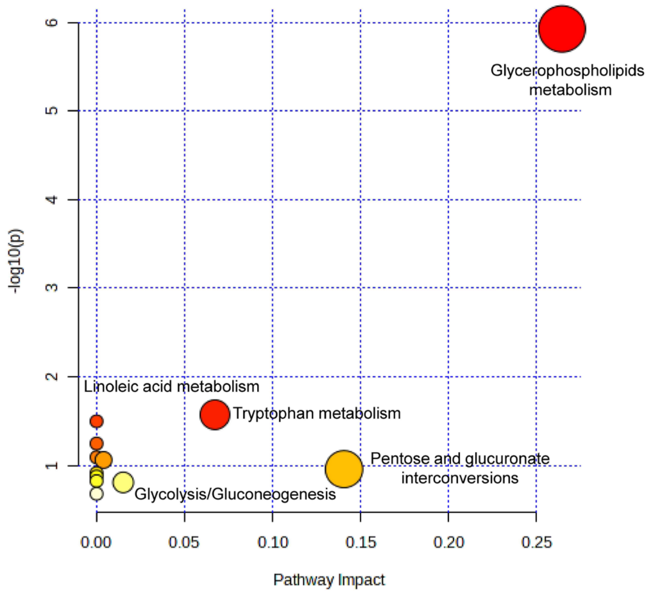

Figure S4. Metabolic pathway analysis of differential plasma metabolites in different group of mice. The y-axis (-$\log_{10}$ (*p*-value)) and x-axis represents the significance of the pathway and pathway impact in differential plasma metabolites in different group of mice, respectively.
